# Taxonomic Variations of Bacterial and Fungal Communities depending on Fermentation Temperature in Traditional Korean Fermented Soybean Food, Doenjang

**DOI:** 10.4014/jmb.2312.12024

**Published:** 2024-01-19

**Authors:** Eunhye Jo, Hyeyoung Lee, Younshil Song, Jaeho Cha

**Affiliations:** 1 Department of Integrated Biological Science, Pusan National University, Busan 46241, Republic of Korea; 2 Food Science & Technology Major, Division of Applied Bioengineering, Dong-eui University, Busan 47340, Republic of Korea; 3 JINA F&C Company, Gyeongju 38033, Republic of Korea; 4 Department of Microbiology, Pusan National University, Busan 46241, Republic of Korea; 5 Microbiological Resources Research Institute, Pusan National University, Busan 46241, Republic of Korea

**Keywords:** Meju, doenjang, fermentation, microbial community, correlation analysis

## Abstract

Meju, a fermented soybean brick, is a key component in soybean foods like doenjang and ganjang, harboring a variety of microorganisms, including bacteria and fungi. These microorganisms significantly contribute to the nutritional and sensory characteristics of doenjang and ganjang. Amplicon-based next-generation sequencing was applied to investigate how the microbial communities of meju fermented at low and high temperatures differ and how this variation affects the microbial communities of doenjang, a subsequently fermented soybean food. Our metagenomic data showed distinct patterns depending on the fermentation temperature. The microbial abundance in the bacterial community was increased under both temperatures during the fermentation of meju and doenjang. *Weissella* was the most abundant genus before the fermentation of meju, however, it was replaced by *Bacillus* at high temperature-fermented meju and lactic acid bacteria such as *Weissella* and *Latilactobacillus* at low temperature-fermented meju. *Leuconostoc*, *Logiolactobacillus*, and *Tetragenococcus* gradually took over the dominant role during the fermentation process of doenjang, replacing the previous dominant microorganisms. *Mucor* was dominant in the fungal community before and after meju fermentation, whereas *Debaryomyces* was dominant under both temperatures during doenjang fermentation. The dominant fungal genus of doenjang was not affected regardless of the fermentation temperature of meju. Strong correlations were shown for specific bacteria and fungi linked to specific fermentation temperatures. This study helps our understanding of meju fermentation process at different fermentation temperatures and highlights different bacteria and fungi associated with specific fermentation periods which may influence the nutritional and organoleptic properties of the final fermented soybean foods doenjang.

## Introduction

Since Koreans have been consuming foods made from fermented soybeans for a long time, there are various manufacturing methods using soybeans in Korea [1]. Doenjang (soybean paste), ganjang (soy sauce), cheonggukjang (soybean sauce), and gochujang (red pepper paste) are known as representative fermented soybean foods in Korea. Unlike cheonggukjang, which is made by fermenting whole soybeans after mashing the steamed beans, doenjang, ganjang, and gochujang use meju as the main ingredient [2]. The traditional Korean meju is produced by soaking, steaming, crushing, and shaping soybeans. After surface drying and fermenting for 1‒2 months, it is used as an ingredient in fermented soybean foods such as doenjang and ganjang [3]. Meju fermentation methods vary depending on the region, manufacturer, and household [1].

A crucial contributing factors in meju fermentation are known to be the fermentation period, the water content of meju, as well as the temperature and humidity of the surrounding environment [3]. Among these factors, the control of fermentation temperature and humidity is widely recognized as a critical factor in promoting the growth of spontaneous microorganisms such as fungi and bacteria [4]. The proper adjustment of these environmental factors is known to stimulate the activation of various enzymes which responsible for the breakdown of carbohydrates, proteins, lipids, and flavonoid glycosides contributing to the nutrition, tastes, flavors, and functionalities in the doenjang and ganjang [5, 6]. Furthermore, precise control of fermentation conditions facilitates the growth of microorganisms essential for meju fermentation while suppressing the growth of toxin-producing microorganisms and the colonization of pathogens [7, 8]. In a previous study, meju fermented at high temperatures and humidity (25–30°C and 80–90%) was found to have a higher concentration of total free amino acids compared to meju fermented at low temperatures and humidity (15–20°C and 40–50%) [9]. Additionally, a study comparing the quality characteristics of three soybean varieties fermented for 10 days at three temperatures showed differences in fungal growth, amino-type nitrogen production, and enzyme activity, all of which were influenced by fermentation temperature [10].

During the manufacturing process to make fermented soybean brick meju, various microorganisms are spontaneously generated from raw materials and the surrounding environment including incubation shelves, air, and rice straw [11, 12]. These microorganisms are known to play a crucial role in the development of organoleptic properties and bioactive functions in the soybean fermented foods. *Bacillus licheniformis* isolated from meju and doenjang has been known to produce floral and nutty aroma compounds [13]. The halophilic lactic acid bacteria *Tetragenococcus halophilus* isolated from meju has been reported to have antioxidant and anti-inflammatory activity [14]. The yeast *Debaryomyces hansenii*, which is isolated from meju, has been reported to produce γ-aminobutyric acid (GABA) indicating a potential role for this genus as a major GABA producer during fermentation [15]. By gathering information on the growth conditions of these fermentation microorganisms and establishing fermentation features, it becomes possible to produce meju harnessing the physiological traits of these microorganisms.

Based on next generation sequencing methods, diverse fermented soybean foods such as meju, doenjang, and ganjang have been investigated for bacterial and fungal communities [16-21]. Although several studies on the microbial community have been conducted using amplicon-based sequencing in soybean fermented foods, metagenomic studies based on distinct manufacturing processes depending on the fermentation temperature have not yet been reported. In this study, we used amplicon-based sequencing to investigate the fermentation characteristics of doenjang made from meju produced at two different fermentation temperatures and analyzed microbial communities. In addition, we performed correlation analysis between different microbial taxa to investigate the bacterial and fungal relationships occurred in meju and doenjang. This study will contribute to the manufacturing traditional fermented soybean foods based on microbial community structures.

## Materials and Methods

### Preparation of Meju and Doenjang and Sampling Strategy

Samples are produced at a manufacturing facility located in Gyeongju, South Korea. The manufacturing process is conducted using traditional methods without intentional microbe inoculation. A total of 40 kg of soybeans (*Glycine max* L. Merrill), Daewonkong cultivar cultivated in Mungyeong providence, Korea, were washed and soaked overnight, steamed for 6 hours, and chilled for 4 h. The steamed soybeans were ground and manufactured into brick shapes measuring 20 cm × 13.5 cm × 10.5 cm and weighing 2.5 kg each. The fermentation of meju involves three steps controlled by temperature and humidity. In the first step, meju bricks were dried at 28°C and 70–80% humidity for 7 days in a fermentation room with rice straw. In the second step, meju bricks were fermented in open-air conditions for 43 days, with temperature ranging from 0–10°C and humidity between 50–60%. In the third step, half of the overall meju bricks was transferred to the fermentation room controlled at 37°C and 40% humidity, designated as high-temperature fermented meju (HTM), while the remaining half underwent fermentation in open-air conditions with temperature about 10°C, labeled as low-temperature fermented meju (LTM). After 2 months, the fermented meju is washed and soaked in a solar salt solution in Korean ceramic pots. The 27.5% brine is mixed with meju bricks in a ratio of 1:3 (w/v). After 2 months, the meju bricks were separated from the brine, mashed equally, and moved to other clean ceramic pots to make doenjang. The doenjang made using HTM and LTM were named as high temperature doenjang (HTD) and low temperature doenjang (LTD), respectively. Both types of doenjang, made from meju fermented in both low and high-temperature conditions, underwent fermentation under the same outdoor conditions from May 2021 to January 2022. They were fermented during the summer, autumn, and winter seasons, with the highest average temperature reaching 29.1°C and the lowest dropping to -6.8°C during that period. The doenjang samples were collected bimonthly.

### Amplicon-Based NGS Library Construction for Bacterial and Fungal Community Analysis

Meju and doenjang samples were stored in a deep freezer until the DNA extraction step. Total genomic DNA was extracted from 0.5 g of samples using the FastDNA SPIN Kit for Soil (MP Biomedicals, USA), following the manufacturer’s instructions. The quality and concentration of extracted total genomic DNA were assessed using Nonodrop (Thermo Fisher Scientific, USA) and checked by electrophoresis using a 1% agarose gel. All the extracted DNA was normalized to 10 ng/μl. The bacterial primer set is for V3/4 regions of 16S rRNA gene. Check the primer info. and the ITS2 region for fungal community is amplified using primer sets ITS86f-ITS4 including Illumina pre-adapter sequence [22-25]. All PCR reactions consisted of 10 μl of 2 × Prime Taq Premix (Genetbio, Republic of Korea), 1 μl of each primer (10 μM), and 1 μl of DNA (5 ng/ μl) with the rest of the 20 μl solution made up of water. The PCR condition included a 95°C step for 3 min, followed by 35 cycles of 95°C for 30 s, 55°C for 30 s, and 72°C for 30 s, with a final 5 min step at 72°C. The amplicons were cleaned up using Expin PCR SV kit (GeneAll Biotechnology, Republic of Korea) and added with index through 2^nd^ PCR. The PCR condition was the same as the first PCR except for the number of cycles, which was 8. The amplicons for each step were confirmed using electrophoresis on a 1% agarose gel and quantified using Nanodrop. The library is pooled in the same quantity and sequenced using he Illumina MiSeq platform, generating 2 × 300 bp paired-end reads at Macrogen (Republic of Korea).

### Bioinformatic processing and Microbial Diversity Analysis

Amplicon sequence variants (ASVs) were generated using the DADA2 package (version 1.10.1) [26]. After checking for fastq sequence using FastQC, entire reads were filtered and trimmed with assigned options; truncLen=c(270,210) for 16S and truncLen=c(200,180) for 18S. Taxonomic assignment for the 16S rRNA gene and 18S ITS region were conducted using the SILVA database (version 138) [27] and UNITE (sh_general_release_ dynamic_all_16.10.2022), respectively. Phyloseq package (version 1.40.0) [28] has been used for representing the relative abundance of microbial communities and estimation of the *κ*-diversity index (Shannon and Chao1) calculated with ASVs. *β*-diversity was confirmed through principal coordinate analysis (PCoA) using the Bray-Curtis dissimilarity distance matrix based on ASVs during fermentation of meju and doenjang.

### Statistical Analysis

To calculate the correlation between dominant bacteria and fungi genera, Spearman correlation analysis was conducted using Hmisc package in R [29]. Cytoscape 3.9.1 was used to visualize the networks [30]. Correlation was adjusted based on Spearman’s correlation coefficients (|r| > 0.75) and statistical significance (*p* < 0.05).

### Sequencing Data Accession Number

The sequence data of the bacterial and fungal metagenomes from this study are available in the NCBI Short Read Archive (SRA) under accession number BioProject PRJNA1040430 and PRJNA1044255, respectively.

## Results

### Differences in Bacterial and Fungal Diversity of Meju and Doenjang Fermented at Low and High Temperatures

The differences in microbial communities during fermentation process at two different temperatures were analyzed. After analyzing the ASV counts, a total of 1,264,783 bacterial 16S rRNA and 2,738,166 fungal ITS reads were obtained from 13 fermented soybean food samples. The average bacterial and fungal community counts were 97,291 and 210,628, respectively. After the steps for filtering/trimming and removing the chimeric sequence, 45.30%and 87.46% of reads were left in the bacterial community and the fungal community, respectively (Table S1).

**Fig. 1:**
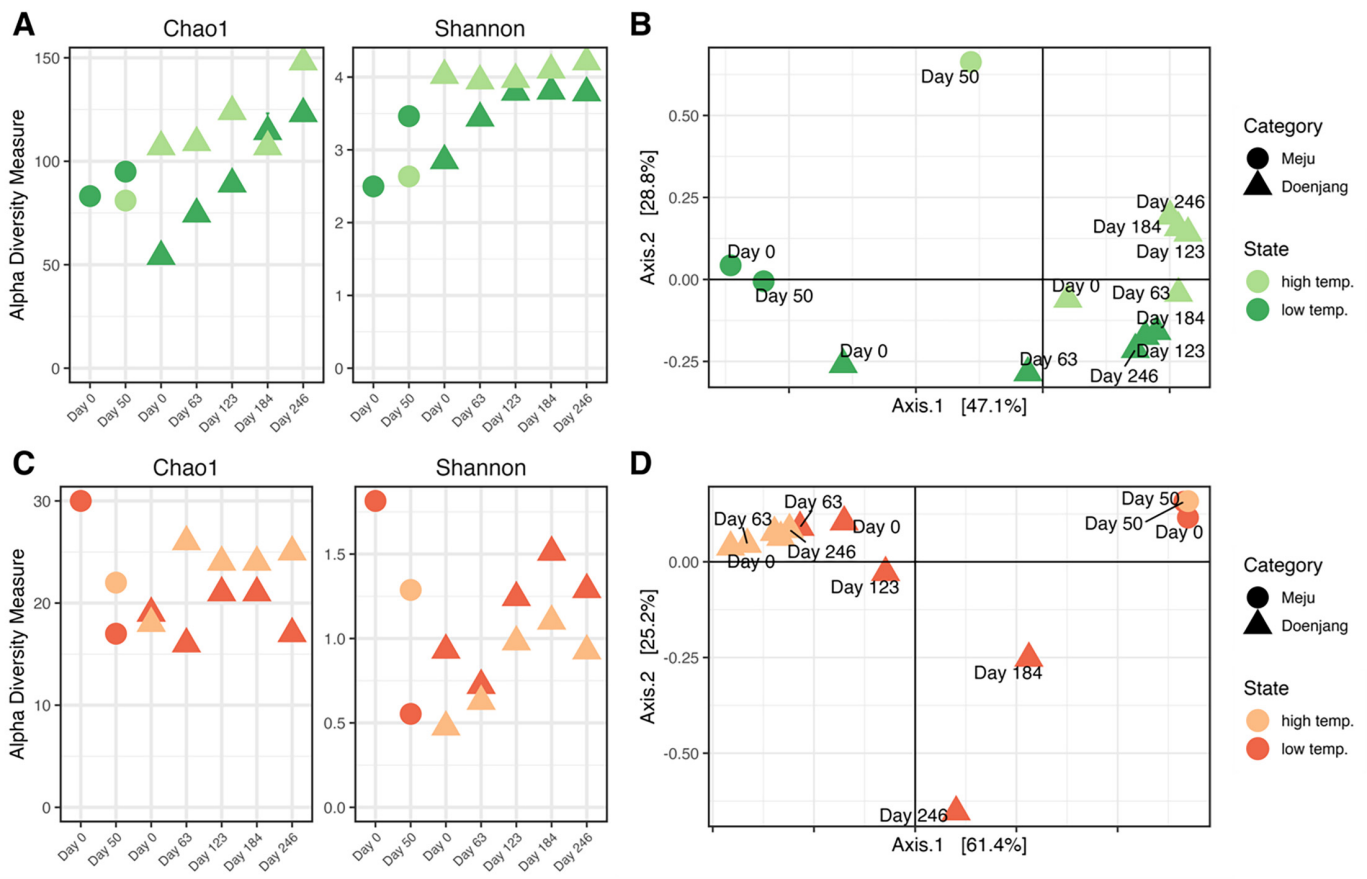
*α*- and *β*-diversity of microbial communities during fermentation. *α*-diversity index (**A**) and principal coordinate analysis for *β*-diversity (**B**) of bacterial community during fermentation is represented with divided meju (circle) and doenjang (triangle). The color of symbols corresponds to the fermentation temperature (light green, high temperature; green, low temperature). *α*-diversity index (**C**) and principal coordinate analysis for *β*-diversity (**D**) of fungal community during fermentation is represented with divided meju (circle) and doenjang (triangle). The color of symbols corresponds to the fermentation temperature (light orange, high temperature; orange, low temperature).

Based on the ASVs, Chao1 and Shannon indices are calculated for microbial abundance and diversity, respectively (Fig. 1A, 1C, and Table S2). Bacterial abundance, represented by the Chao1 index, in the final meju was higher under the low temperature condition (95.0) than under the high temperature condition (81.0). The abundance and diversity of meju increased consistently regardless of the fermentation temperature. In more detail, HTD showed a stable diversity index from the start to the final period, although LTD showed an increase in diversity until the end.

In the fungal community, the initial abundance and diversity, which were higher at the start of the fermentation (Day 0), declined by the end of the period (Day 50). The decrease in diversity was expected due to the appearance of dominant species. The abundance of the fungal community remained stable until the doenjang fermentation. Otherwise, the diversity of the fungal community increased, with LTD having a higher diversity than HTD.

PCoA analysis based on the Bray-Curtis matrix also indicated that a structural differences of microbial communities between samples depending on the fermentation temperature of meju (Fig. 1B and 1D). Whereas there were notable distinctions in the bacterial community of meju, they gradually transitioned into a more consistent composition as the fermentation of doenjang proceeded. Additionally, the variation at high temperatures was lower than that at low temperatures; 28.8% compared to 47.1%. Within the fungal community, the microbial structural differences in meju emerged by the temperature variation were relatively minor, whereas in the doenjang samples, the direction and gradient of compositional change were noticeable. And the microbial variation at low temperature samples was markedly less than that of the high temperature samples; 25.2% and 61.4%, respectively. Consequently, the fermentation temperature of meju did not appear to have a significant impact on the composition of the fungal community of meju, but it seems to influence the occurrence and composition of the fungal community of doenjang.

### Differences in Bacterial and Fungal Communities of Meju and Doenjang Fermented at Low and High Temperatures

**Fig. 2:**
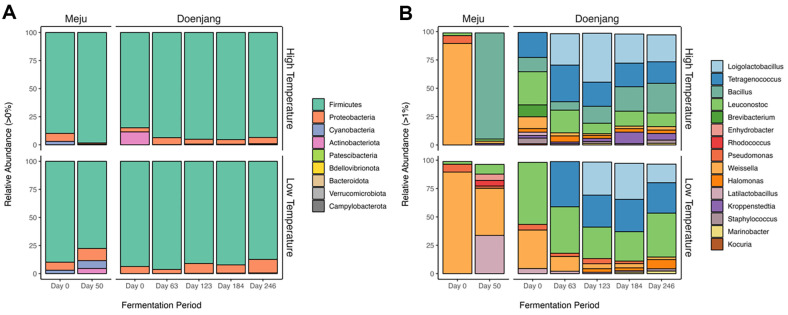
Relative abundance of bacterial communities of meju and doenjang at phylum level (**A**) and genus level (**B**), obtained by 16S rRNA ASVs. In the genus level, 1% cut-off was applied to represent the dominant genera at each sample.

To identify the microbial communities produced during fermentation, the ASVs from the 16S rRNA and ITS sequencing reads were classified at the phylum and genus levels. The overall number of ASVs in bacterial communities was 532, with 526 (98.87%) annotated ASVs in phylum level and 388 (72.93%) in genus level. At the bacterial communities, 9 phyla and 73 genera were identified. After applying a 1% cut-off at the genus level, only 15 genera remained (Fig. 2). *Weissella* (89.6%) was the most abundant bacterial genera followed by *Pseudomonas* (6.9%), *Leuconostoc* (2.4%) on day 0 of meju fermentation. At the LTM, the dominant genera were *Weissella* (41.5%), *Latilactobacillus* (33.7%), *Leuconostoc* (8.6%), *Enhydrobacter* (5.6%), *Rhodococcus* (5.0%), and *Pseudomonas* (2.0%). Conversely, meju fermented at high temperatures (HTM) using the same day 0 meju changed into a dominant *Bacillus* (93.6%) followed by *Weissella* (2.0%), and *Leuconostoc* (1.9%) on day 50 of fermentation.

Since doenjang is made by putting fermented meju in salt water, diverse microorganisms derived from the salt water are introduced. In LTD, it was found that bacteria present in the meju, including *Leuconostoc* and *Weissella*, were dominant, and the settlement of microorganisms derived from salt water was processed slowly. On the other hand, in the HTD, the proportion of *Bacillus* was greatly reduced, and microorganisms derived from salt water rapidly settled. *Tetragenococcus*, one of the critical microorganisms for doenjang fermentation, is a halophilic gram-positive lactic acid bacterium [31]. Along with the other lactic acid bacteria, it is known that *Tetragenococcus* contributes to the sensory features and nutritional functionalities of fermented foods. By accumulating information on microbial structures associated with fermentation temperature of meju and its impact on sensory attributes, it becomes possible to determine the required temperature and fermentation duration to achieve the desired sensory properties in fermented food production.

**Fig. 3:**
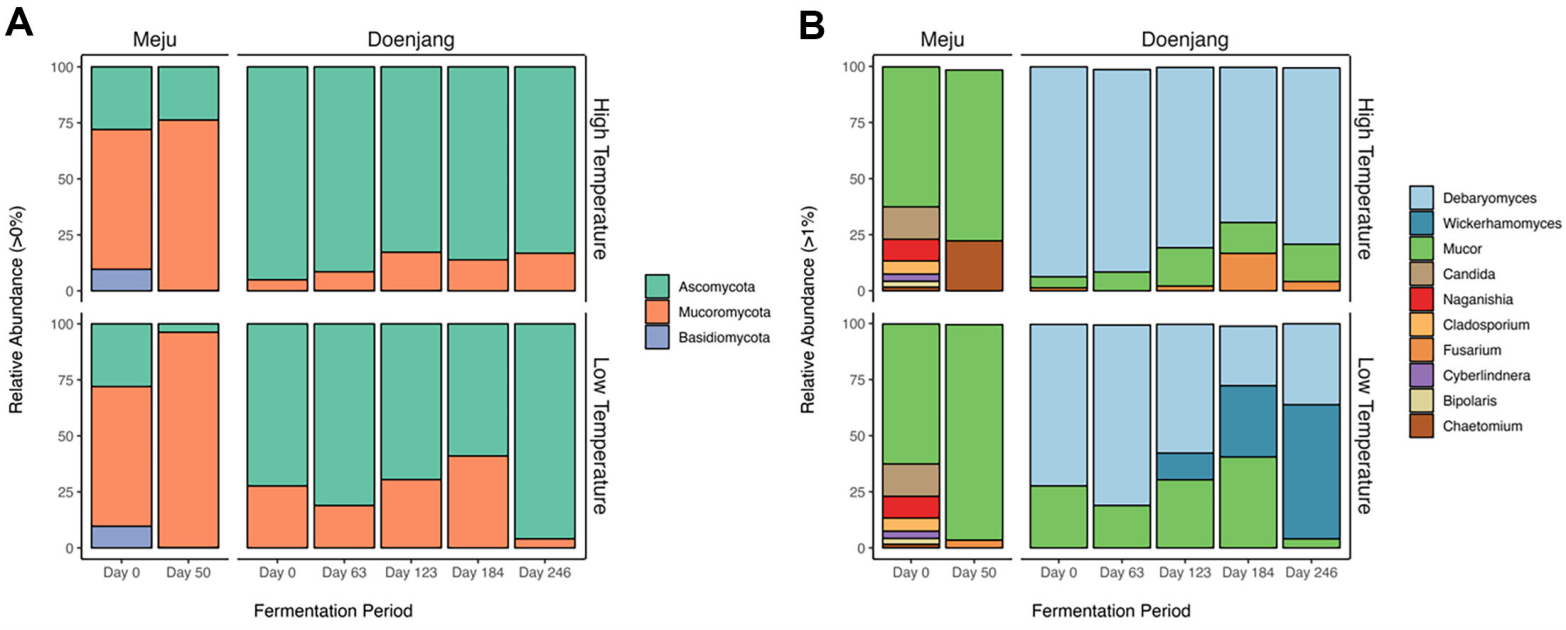
Relative abundance of fungal communities of meju and doenjang at phylum level (**A**) and genus level (**B**), obtained by ITS ASVs. In the genus level, 1% cut-off was applied to represent the dominant genera at each sample.

The overall number of ASVs in fungal communities was 114, and annotated ASVs at the phylum and genus levels were 111 (97.37%) and 107 (93.86%), respectively. In the phylum level, *Mucor*omycota (62.4%), Ascomycota (28.0%), and Basidiomycota (9.7%) appeared on day 0 of meju fermentation, and by the end of meju fermentation, *Mucor*omycota accounted for 76.1% in HTM and 96.0% in LTM, respectively (Fig. 3). However, within the doenjang samples, it was observed that Ascomycota dominated, accounting for a minimum of 58.96%, an average of 75.57%, and a maximum of 95.94% in the LTD, while in the HTD, it was confirmed to occupy a minimum of 82.73%, an average of 87.71%, and a maximum of 95.03%. At the genus level, it was confirmed that unique and distinct fungal genus appeared specifically in response to temperature variations. Halophilic yeast genus *Debaryomyces* derived from salt water was commonly dominant, but *Fusarium* appeared in HTD (16.70%) and *Wickerhamomyces* appeared in LTD (31.75%) in the late stage of fermentation (Day 184). *Debaryomyces* is an indigenous yeast commonly identified not just in ganjang and doenjang but also in cheese and fermented meat products. There have been reports suggesting its potential for aroma compound using *Debaryomyces* sp. isolated from ganjang and its utility as probiotics [32]. *Fusarium* is a filamentous fungus commonly existing in the natural environment and some species are known to produce mycotoxins, which are recognized as harmful to both wildlife and livestock. *Wickerhamomyces*, a yeast found in the later stages of LTD, has been gradually increasing. The yeast has been reported to exhibit growth-inhibiting activity against harmful microorganisms when isolated from fruits, fermented beverages, and has been reported for its characteristics in Korean traditional brewing [33]. These results suggest that the fermentation temperature of meju can influence specific fungi during doenjang fermentation.

### Correlation between Bacterial and Fungal Genera of Meju and Doenjang Fermented at Low and High Temperatures

**Fig. 4:**
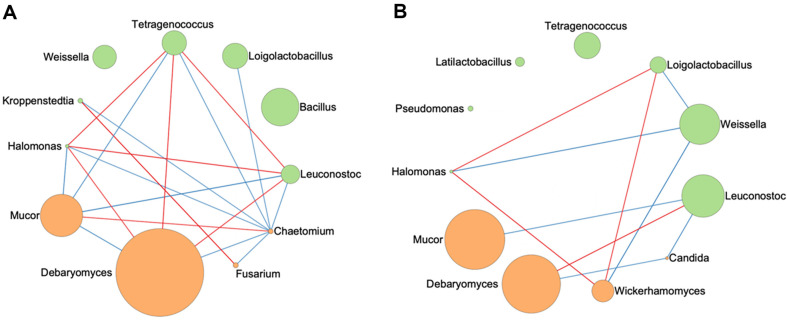
Spearman correlation analysis between the seven most abundant bacteria genera and the four most abundant fungal genera at high (**A**) and low (**B**) fermentation temperatures. Only statistically significant correlations are displayed (|r| > 0.75, *p* < 0.05). Correlation coefficients are colored red (positive correlation) to blue (negative correlation). Bacterial and fungal genera are colored in green and red, respectively.

To further evaluate the potential associations between dominants bacteria and fungi in meju and doenjang, the correlation between 7 bacterial genera and 4 fungal genera was analyzed using Spearman’s correlations. The results were visualized using correlation coefficients and relative abundance (Fig. 4). Under high temperature conditions, nine of the 11 genera (*Loigolactobacillus*, *Tetragenococcus*, *Leuconostoc*, *Kroppenstedtia*, *Halomonas*, *Debaryomyces*, *Mucor*, *Fusarium*, and *Chaetomium*) demonstrated significant correlations with other genera, including five bacteria and four fungi. This resulted in 11 negative and 8 positive correlations (|ρ| ≥ 0.75, *p* < 0.05). However, under the low temperature conditions, eight of the 11 genera (*Leuconostoc*, *Weissella*, *Loigolactobacillus*, *Halomonas*, *Mucor*, *Debaryomyces*, *Wickerhamomyces*, and *Candida*) showed significant correlations, resulting in 6 negative and 4 positive correlations (|ρ| ≥ 0.75, *p* < 0.05). While *Bacillus* did not show a significant correlation with any genus under the high temperature conditions, *Tetragenococcus* was positively correlated with *Debaryomyces*, *Halomonas*, and *Leuconostoc* and negatively correlated with *Chaetomium* and *Mucor*. On the other hand, *Tetragenococcus* did not exhibit any significant correlation under the low temperature conditions. Significant correlations observed during the fermentation of meju and doenjang provide potentials suggesting the co-occurrence of microorganisms [34]. The high fermentation temperature of meju is supposed to have influenced the metabolic interactions of *Tetragenococcus*, a predominant species in doenjang, with other microorganisms. These results suggest the fermentation temperature of meju can influence microbial interactions among the main bacteria and fungi.

## Discussion

The traditional meju produced in Korea is naturally fermented soybean brick which resides in various bacteria and fungi derived from the surrounding environments. The nutritional and functional properties of fermented soybean paste doenjang produced by further fermentation of meju are influenced by the autochthonous microorganisms present in meju, and it is known that the distribution and structure of this microbial community is greatly affected by fermentation temperature [4, 35]. In this study, the difference in bacterial and fungal communities in doenjang, made from two types of meju fermented at high and low temperatures, were analyzed using amplicon-based metagenomics. Additionally, the interactions between the bacterial and fungal groups were examined through correlation analysis of predominant bacteria and fungi.

Within the bacterial community, *Bacillus* was dominant in high temperature-fermented meju (HTM), while *Weissella*, *Leuconostoc*, and *Latilactobacillus* were dominant in low temperature-fermented meju (LTM). Previously, it has already been reported that *Bacillus* and lactic acid bacteria were major bacterial groups in meju fermentation, and that their abundance varied depending on the manufacturers [17, 36]. In our study, the dominant bacteria during meju fermentation were gradually replaced by *Leuconostoc*, *Logiolactobacillus*, and *Tetragenococcus* during progress of doenjang fermentation at both temperatures. It is thought that the appearance of these halotolerant bacteria during doenjang fermentation progress, regardless of the fermentation temperature of meju, is because the salt water added during the manufacturing process provides favorable conditions for the survival of these bacteria. Previous studies also confirmed that *Tetragenococcus* was the dominant bacteria in doenjang fermentation regardless of fermentation conditions [37]. Nevertheless, a greater diversity of bacterial taxa during the progress of doenjang fermentation in HTD compared to LTD implies that the relative abundance of *Bacillus* and lactic acid bacteria, known as major contributors for organoleptic properties in fermented foods [38, 39], found in meju is highly influenced the bacterial group during doenjang fermentation. The predominance of *Bacillus* in HTD is thought to have contributed to the decomposition of carbohydrate and protein components of meju, thereby aiding the colonization or settlement of bacteria introduced with the salt water. Various carbohydrate decomposition activities and high protease activities of *Bacillus* isolated from meju have been reported [40-42]. This, in turn, would affect specific flavors and functional properties of the fermented food, doenjang.

Fungal community analysis revealed that *Mucor*omycota was abundant in both HTM and LTM, however, Ascomycota was most abundant through in doenjang fermentation, which is in accordance with previous reports [37]. At the genus level, *Debaryomyces* was commonly dominant, but *Fusarium* and *Wickerhamomyces* as minor genera were appeared in HTD and LTD in the late stage of fermentation, respectively. *Debaryomyces* is an indigenous yeast commonly identified in soybean fermented foods, ganjang and doenjang, as well as dairy and meat fermented foods [43, 44]. It has been reported as fungus which affects aroma properties in fermented foods. *D. hansenii* isolated from ganjang is known to produce isoamyl alcohol and phenylethyl alcohol in YPD media, resulting an alcoholic, fruity and sweet flavor [32]. *Wickerhamomyces* has been reported to exhibit growth-inhibiting activity against harmful microorganisms when isolated from fruits, fermented beverages, and has been reported for its characteristics in Korean traditional brewing [33]. Therefore, the bacterial and fungal community analyses implied that the fermentation characteristics of doenjang might vary due to differences in the microbes responsible for doenjang fermentation depending on the fermentation temperature of meju. These results can contribute to the production of specifically intended doenjang by traditional soybean food manufacturers.

The correlation analysis between microbial taxa during meju and doenjang fermentation clearly showed that the microbial interactions is largely different depending on low and high fermentation temperature. More positive and negative correlations were identified between bacterial and fungal taxa in high temperature fermentation than in low temperature fermentation. These reliable correlations can provide evidence to hypothesize suggesting that there may have been metabolic interactions under the high temperature among microorganisms in actual fermentations [34]. The correlation analysis of bacterial community in fermented foods Kinema and Sufu provided valuable insights for potential symbiotic and antagonistic relationships among the microorganisms under the condition [45, 46]. It is thought that *Bacillus*, which dominates at high-temperature meju, decomposes the nutrients using fibrinolytic enzyme and proteolytic enzymes, affecting the growth of other microorganisms [41, 42]. These activities are supposed to impact to the early colonization of other microorganisms derived from salt water such as *Tetragenococcus* and *Logiolactobacillus*. Furthermore, in a study investigating bacterial diversity and physicochemical properties of low-salt fermented shrimp paste, *Tetragenococcus* exhibited a positive correlation with the physicochemical properties under conditions resulting from high-temperature fermentation [47]. It is inferred that these conditions contribute to the enhancement of the food fermentation process. In conclusion, our study indicates that the microbial communities in doenjang are significantly influenced by the fermentation temperature of meju, potentially impacting the overall characteristics of doenjang fermentation. These findings establish a scientific foundation for tailoring the production of fermented foods with distinct flavors or functionalities through the precise control of fermentation temperature conditions.

## Supplemental Materials

Supplementary data for this paper are available on-line only at http://jmb.or.kr.
