# Supplementary material for: Taxonomic Variations of Bacterial and Fungal Communities depending on Fermentation Temperature in Traditional Korean Fermented Soybean Food, Doenjang
Source: J Microbiol Biotechnol. 2024 Jan 19;34(4):863–70. doi: 10.4014/jmb.2312.12024 (PMC11091663; doi:10.4014/jmb.2312.12024)
Supplement: Supplementary file 1 [file jmb-34-4-863-supple.pdf]

Supplementary Table S1.

Samples	Temperature	Day	Bacterial community				Fungal community			
			input	filtered	merged	HQR	input	filtered	merged	HQR
Soybean brick		0	123234	95101	92469	65248	312396	283667	275637	255715
Meju	low	50	147115	96660	93080	59921	356832	332296	330520	323961
	high	50	151654	97989	96337	70645	262175	241503	239223	219901
Doenjang	low	0	82165	75880	74000	42390	145477	141774	137158	133513
		63	73813	68242	66127	33110	165820	162115	156980	154659
		123	82172	75525	73425	37029	182906	176003	169934	163471
		184	82747	75522	73650	40236	233814	223084	213096	208679
		246	86190	78862	76537	37797	169891	161692	151558	131768
	high	0	80858	73165	70240	29684	163392	157527	152761	151933
		63	85104	78200	76172	34485	186221	182322	177679	176317
		123	79627	72661	70635	32748	193370	175989	170579	167458
		184	89484	80948	78826	36811	167216	147620	135260	131681
		246	100620	91302	89234	52790	198656	186806	177940	175742
Sum			1264783	1060057	1030732	572894	2738166	2572398	2488325	2394798
Mean			97291	81543	79287	44069	210628	197877	191410	184215
%			100.00	83.81	81.49	45.30	100.00	93.95	90.88	87.46

HQR: high-quality reads

Supplementary Table S2.

Samples	Temperature	Day	Bacterial community				Fungal community			
			Observed	Chao1	Shannon	Simpson	Observed	Chao1	Shannon	Simpson
Soybean brick		0	83	83.2	2.498	0.863	89	89.0	2.565	0.843
Meju	low	50	95	95.0	3.463	0.953	18	18.0	0.554	0.220
	high	50	81	81.0	2.634	0.879	23	23.0	1.288	0.620
Doenjang	low	0	54	54.0	3.436	0.953	20	20.0	0.931	0.476
		63	73	74.5	3.436	0.969	16	16.0	0.722	0.369
		123	89	89.0	3.792	0.969	21	21.0	1.242	0.614
		184	105	114.3	3.807	0.961	22	22.0	1.517	0.719
		246	123	123.0	3.783	0.919	17	17.0	1.290	0.627
	high	0	107	107.0	4.022	0.973	18	18.0	0.475	0.188
		63	109	109.0	3.947	0.975	26	26.0	0.627	0.253
		123	124	124.0	3.967	0.971	25	25.0	0.983	0.404
		184	107	107.0	4.095	0.979	24	24.0	1.103	0.521
		246	148	148.0	4.209	0.979	26	26.0	0.927	0.407
